# CRISPR/Cas9-mediated modulation of splicing efficiency reveals short splicing isoform of Xist RNA is sufficient to induce X-chromosome inactivation

**DOI:** 10.1093/nar/gkx1227

**Published:** 2017-12-09

**Authors:** Minghui Yue, Yuya Ogawa

**Affiliations:** 1 Division of Reproductive Sciences, Division of Developmental Biology, Perinatal Institute, Cincinnati Children's Hospital Medical Center, Cincinnati, OH 45229, USA; 2 Department of Pediatrics, University of Cincinnati College of Medicine, Cincinnati, OH 45267, USA

## Abstract

Alternative splicing of mRNA precursors results in multiple protein variants from a single gene and is critical for diverse cellular processes and development. *Xist* encodes a long noncoding RNA which is a central player to induce X-chromosome inactivation in female mammals and has two major splicing variants: long and short isoforms of Xist RNA. Although a differentiation-specific and a female-specific expression of Xist isoforms have been reported, the functional role of each Xist RNA isoform is largely unexplored. Using CRISPR/Cas9-mediated targeted modification of the 5′ splice site in *Xist* intron 7, we create mutant female ES cell lines which dominantly express the long- or short-splicing isoform of Xist RNA from the inactive X-chromosome (Xi) upon differentiation. Successful execution of CRISPR/Cas-based splicing modulation indicates that our CRISPR/Cas-based targeted modification of splicing sites is a useful approach to study specific isoforms of a transcript generated by alternative splicing. Upon differentiation of splicing-mutant *Xist* female ES cells, we find that both long and short Xist isoforms can induce X-chromosome inactivation normally during ES cell differentiation, suggesting that the short splicing isoform of Xist RNA is sufficient to induce X-chromosome inactivation.

## INTRODUCTION

Alternative splicing of mRNA precursors is widespread in multicellular eukaryotes, especially in higher vertebrates (1,2). In multicellular eukaryotes, alternative splicing is more common than in unicellular eukaryotes in which most of genes are intron-less or very short introns and alternative splicing is rarely found. The total number of genes is not radically different between vertebrates and invertebrates, but the numbers of alternative spliced genes and the number of variants are higher in vertebrates, suggesting that alternative splicing could be related with the complexity of species. For example, in humans, ∼98% of multi-exon genes undergo alternative splicing (3). Significant expansion of the proteome generated through alternative splicing from limited numbers of genes provides diverse regulatory functions for proteins such as a tissues-specific and developmental stage-specific functions (4).

*Xist* encodes a long noncoding RNA and is required for X chromosome inactivation (XCI) by which one of the two X-chromosomes is transcriptionally silenced in female mammals (5–8). During XCI, Xist RNA highly expressed from the inactive X-chromosome (Xi) recruits various chromatin modifying enzymes to the Xi and induces chromosome-wide epigenetic modifications (9,10). Disruption of *Xist* expression results in failure of female embryonic development or induction of cancer in females (11,12), indicating the critical role for *Xist* throughout the female life cycle. *Xist* is transcribed into a variety of different isoform transcripts through differentiation-specific transcription start sites (13), alternative polyadenylation sites (14,15), and alternative splicing (16). Although there are various isoforms of Xist RNA, the specific functions of each remain unexplored.

A previous report using tetracycline-inducible *Xist* mutant transgenes integrated in X-linked *Hprt* locus in male ES cells demonstrated that repeat A located at the 5′-end of Xist RNA is essential for X-linked gene silencing, and functionally redundant elements for Xist RNA localization are dispersed across the rest of *Xist* region (17). In this assay, *Xist* mutant transgene lacking the 3′-half of Xist RNA including exon 7 still exhibits normal Xist RNA localization and induction of X-linked gene silencing. Using the *Xist* transgene assay, however, the role of *Xist* elements for XCI can be addressed only at the early stage of XCI, since inactivation of the single male X-chromosome leads to cell death. Thus, the role of the 3′-half region of Xist RNA including exon 7 in XCI has been overlooked until recently. Several papers using *Xist* mutant female cells have shown that the critical *Xist* elements/regions for XCI reside across Xist RNA (18–22). Our recent study demonstrated that exon 7 of long splicing isoform of Xist RNA is essential for stable Xist RNA localization on the Xi and harbors one of the two major binding region for heterogeneous nuclear ribonucleoprotein U (hnRNP U) protein required for anchoring Xist RNA to the Xi (20,23). The short splicing isoform of Xist RNA, which loses a large part of exon 7 present in the long splicing isoform of Xist RNA, is reported as a female-specific isoform of Xist RNA (16). Since the short splicing isoform of Xist RNA loses one of two major hnRNP U binding regions present in exon 7 of the long splicing Xist RNA isoform, we sought to address whether the short splicing Xist isoform is capable of inducing XCI.

To investigate the function of specific splicing isoforms of Xist RNA, modification of the 5′ and 3′ splice sites or deletion of the intron is one potential approach. Modulation of splicing efficiency by altering the consensus sequence for splicing at the 5′ and 3′ splice sites may result in a dominant expression of a specific isoform of transcripts without disturbing the majority of the genomic sequence. Instead of traditional gene targeting, the CRISPR (clustered regularly interspaced short palindromic repeat)/Cas (CRISPR-associated) system derived from microbes has widely been used as a tool to modify genomic DNA sequence, because it is easy to design and efficient for genome editing in a variety of systems (24,25). Here we show an efficient strategy to modulate *Xist* intron 7 splicing using the CRISPR/Cas9 system. We targeted *Xist* intron 7 in mouse female ES cells to generate cell lines dominantly expressing the short- or long-splicing isoform of Xist RNA. To our surprise, the dominant expression of the short splicing Xist isoform induced by mutations at the 5′ splice site did not affect Xist RNA localization nor X-linked gene silencing. We discuss the implications of these results to understand the potential role of the short splicing isoform of Xist RNA in XCI.

## MATERIALS AND METHODS

### Cell culture

Wildtype J1 male and 16.7 female ES cells and their derivatives *Tsix*-truncated J1 male and 16.7 female ES cells have been described previously (20,26–28). *Tsix*-truncation mutant female ES cells expressing FLAG-HA-hnRNP U from endogenous hnRNP U loci has been described previously (20). ES cells were cultured in ES media with 2i inhibitors and induced differentiation by the embryoid body procedure (20). To generate MEF, female *Mus musculus* 129S1/SvlmJ mouse (Jackson Laboratory) was crossed with male *M. musculus castaneus* mouse (CAST/EiJ, Jackson Laboratory). MEF cells were established from E12.5 mouse embryo of resulting progeny.

### ES cell targeting with CRISPR/Cas9 system

For *Xist* intron 7 targeting, synthetic forward and reverse primers were annealed and inserted to the modified pX459ver2 plasmid with the sgRNA^(F+E)^ mutation at BbsI (21,29,30). ssODNs for *Xist* splicing-enhanced or -repressed were used for the homology directed repair (HDR)-mediated modification for SXT or LXT *Xist* splicing mutations, respectively. Primer information is listed in Supplementary Table S1.

For *Tsix* intron 2 targeting, SpCas9 in the modified pX459ver2 plasmid was replaced by high specificity eSpCas9(1.1) (31), yielding pX459Me. In addition, we introduced VQR mutations to eSpCas9(1.1) for NGA PAM recognition (32). A synthetic tRNA promoter primer pair for sgRNA without 5′-G to replace U6 promoter (33) and a primer pair for sgRNA were annealed and inserted to pX459Me(+VQR) plasmid at PciI-BbsI site. ssODNs STsT were used for HDR-mediated modification to enhance incorporation of *Tsix* exon 3.

Transfection and selection for CRISPR/Cas9-mediated genome editing using ssODN were described previously (20). Genomic PCR primers (X7) used to identify mutant clones in Figure 2C and Supplementary Figure S3C are: *Xist*, Xist-AI7-SD-F and Xist-AI7-SD-R; and *Tsix*, Tex2-F1 and Tint3-R1, respectively.

### Generation of female ES cells expressing long or short splicing Xist isoform by gene targeting

Bacterial recombineering system was used to construct the vector for targeting the 5′ and 3′ splice sites of *Xist* intron 7 (34). For targeting the 5′ splice site of *Xist* intron 7, the left- and right-arm adaptors for bacterial homologous recombination were annealed and inserted into pBS-2xLoxP-Zeo at XhoI/HindIII and EcoRI/NotI sites (20), respectively. For targeting the 3′ splice site of *Xist* intron 7, the left- and right-arm adaptors were inserted into pBS-2xLoxP-Zeo, at XhoI/HindIII and EcoRI/NotI sites, respectively. BstZ17I/SpeI splice acceptor (SA)-internal ribosome entry site (Ires)-puromycin resistance gene (Puro)-truncated thymidine kinase (ΔTK)-tandem poly(A) signals (tpA) cassette from pGEM-SAIres-PuroΔTK-tpA or SA-Ires-hygromycin resistance gene (Hyg)-ΔTK-tpA cassette from pGEM-SAIres-HygΔTK-tpA was inserted at PmlI/NheI between two LoxP sites, yielding the targeting vector for bacterial recombination for the 5′ or 3′ splice sites of *Xist* intron 7, respectively. pBS-sx16delL containing a 9.8 kb fragment from *Xist* exon 1 to exon 7 (chrX: 103 464 270–103 474 034 in GRCm38/mm10, UCSC genome browser) was transfected into SW106 strain and targeted by XhoI-NotI fragment of the bacterial targeting vector, generating the 5′ splice site targeting vector for mouse ES cells. pBS-sx16delL2 containing a 8.4 kb fragment from *Xist* exon 7 to downstream region (chrX: 103 457 842–103 466 223 in GRCm38/mm10, UCSC genome browser) and XhoI-NotI fragment of the bacterial targeting vector were used for generating the 3′ splice site mouse targeting vector. The *Xist* intron 7 targeting vectors linearized by SalI were used for transfection of mouse ES cells by electroporation.

For transfection of targeting construct into mouse ES cells, 2 × 10^7^ ES cells and 40 μg of linearized mouse targeting vector were used for electroporation using the Bio-rad Gene Pulser Xcell and 0.4 cm gap cuvette at 240 V, 500 μF setting. The transfected mouse ES cells were cultured without selection drug for 24 hours and then cultured with 250 μg/ml Hygromycin or 2 μg/ml Puromycin for 7–8 days. To remove selection marker by Cre-loxP recombination, cells were transfected with Cre-expression vectors and were selected by 0.2 μM Fialuridine (FIAU, Sigma-Aldrich) for 7–8 days to isolate TK-negative colonies. Isolated individual ES colonies were screened by genomic PCR using primer sets (the 5′ splice site targeting, 7.2TST5-F and UptpA-F for 5′-end, SA-R, and 7.2TSTIn-R for 3′-end; 3′ splice site targeting, 7.2TSTIn-F and UptpA-F for 5′-end, SA-R and 7.2TST3-R for 3′-end; *Tsix* targeting, TST-F and SA-R for 5′-end, TST-R and UptpA-F for 3′-end) as described in Supplementary Figure S2.

### Reverse transcription PCR (RT-PCR)

RT-PCR were performed according to Yamada *et al.* (20). For reverse transcription and quantitative real time PCR (qRT-PCR) analysis of each *Xist* splicing mutant, two independent mutant cell lines were used. The qRT-PCR data is an average of three independent differentiation experiments per each mutant cell line. Briefly, total RNA was extracted using RNAzol RT (Molecular Research Center) as per the manufacturer's instructions. DNA contamination was eliminated from 2 μg of total RNA using 0.5 units TURBO DNase (Invitrogen) at 37°C for 1 hour. 0.4 μg RNA was used for cDNA synthesis with gene-specific reverse primers using Maxima H Minus Reverse Transcriptase (Thermo Scientific) at 50°C for 30 min according to the manufacturer's instructions. As a minus RT control, reaction without Maxima H Minus Reverse Transcriptase was performed in parallel to ensure specificity of the qRT-PCR. cDNA was 5-fold diluted with H_2_O and stored in –20°C.

Real-time PCR reactions were performed by adding 0.5 μl cDNA and 0.5 μM forward and reverse primers to Fast SYBR Green Master Mix in StepOnePlus Real-Time PCR System (Applied Biosystems). All real-time PCR was done in triplicate with the conditions as followed: 1 min at 95°C (1×), followed by 15 s at 95°C, 30 s at 58–62°C (See Supplementary Table S1) (40×). A melt curve from 60 to 95°C was run to ensure only one specific product was amplified. Primer pairs used in qRT-PCR was previously described except for forward primer to detect short Xist isoform (Supplementary Table S1) (20): 129 allele-specific *Xist* (X1–3), Xist^129^-E1–3-F and Xist^129^-E1–3-R; 129 allele-specific *Pgk1*, Pgk1^129^-F and Pgk1^129^-R; 129 allele-specific *Mecp2*, Mecp2^129^-F and Mecp2^129^-R; *Gapdh*, Gapdh-F and Gapdh-R; long Xist isoform (XL), XiI7LRT-F and XiI7LRT-R; short Xist isoform (XS), XiI7SRT-F2 and XiI7SRT-R.

For RT-PCR analysis of *Tsix* splicing mutant, PCR reactions were performed using 1 μl cDNA and 0.5 μM forward and reverse primers in total 20 μl Maxima Hot Start PCR Master Mix (Thermo Scientific). PCR reaction was performed with the conditions as followed: 4 min at 95°C (1×), followed by 30 s at 95°C, 30 s at 58°C, and 20 s at 72°C (28×) for *Tsix* or 10 s at 72°C (24×) for *Gapdh*. Primer pairs used in PCR: *Tsix* exons 2–4, Tex2-F1 and Tex4-R1; *Gapdh*, Gapdh-F and Gapdh-R.

### Immuno-FISH and immunofluorescence

Immuno-FISH was performed as described (21,35). Antibodies used in this study are: anti-H3K27me3 (MABI #0323, 1:1000 dilution and Cell Signaling #9733, 1:1000), anti-H2AK119Ub (Cell Signaling #8240, 1:1000), anti-H4K20me1 (Active motif #39727, 1:5000), anti-ASH2L (Bethyl Laboratories A300-107A, 1:400).

### UV-crosslinking RNA immunoprecipitation (RIP) analysis

UV-Crosslinking RIP was performed following a previously described procedure (20). qRT-PCR was performed using the primers listed in Supplementary Table S1.

## RESULTS

### Short splicing isoform of Xist RNA is expressed in both male and female ES cells

**Figure 1.**
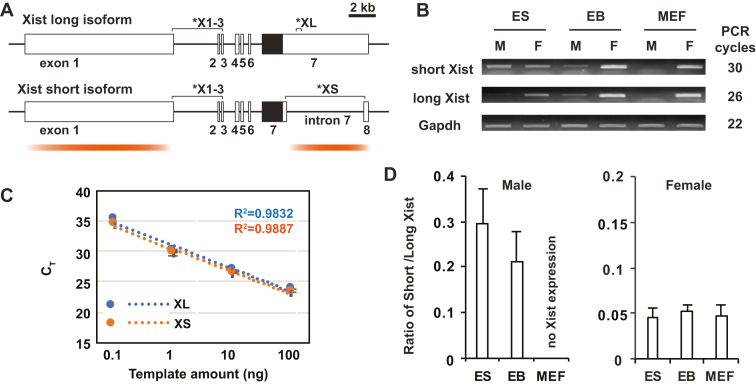
Expression of short and long splicing isoform of Xist RNA in male and female cells. (**A**) Schematics of short and long splicing isoform of Xist. White and black boxes indicate *Xist* exons and repeat E, respectively. The positions of primer pairs are shown as asterisks: X1–3, total *Xist* expression; XL and XS, long and short splicing isoform of *Xist* expression, respectively. Two major hnRNP U binding regions in Xist RNA are shown by orange gradient lines. (**B**) RT-PCR of short and long splicing isoform *Xist* expression in *Tsix* mutant ES cells and embryoid bodies (EB), and wild-type mouse embryonic fibroblast (MEF) cells derived from *Mus musculus* 129S1/SvlmJ and *Mus musculus castaneus* (CAST/EiJ) mating. M, male; F, female. (**C**) Standard curve for short and long Xist splicing isoform-specific primer pairs, XL and XS shown in (A). X-axis, relative amount of serial dilutions of input genomic DNA (100 ng to 100 pg) extracted from heterozygous *Xist* intron 7 deletion mutant female ES cells; y-axis, the C_T_ number measured by real time PCR. (**D**) Ratio of short and long Xist splicing isoform expressed in male and female ES, EB and MEF cells. The mean ± SD from three independent experiments is shown.

Compared with the long splicing isoform of Xist RNA, the short splicing isoform of Xist RNA lacks a 5668 nt-length fragment located downstream of repeat E at the 5′-end of *Xist* exon 7 (Figure 1A). Although a previous report showed that the short splicing isoform of Xist RNA is transcribed in female cells (16), the presence ratio remains unclear between short and long splicing Xist RNA isoforms in undifferentiated embryonic stem (ES) and differentiated female cells. To determine the short/long Xist isoform ratio, we designed short and long splicing isoform-specific primer pairs for quantitative RT-PCR (qRT-PCR) (XS and XL in Figure 1A). Male and female ES cells used in this work contain a mutation in *Tsix*, a *Xist* antagonist, which results in higher *Xist* expression in undifferentiated male and female ES cells and leads to non-random X-inactivation in female cells upon induction of X-inactivation (20,26). As previously reported, *Xist* exhibited sex-specific dynamic expression patterns during X-chromosome inactivation (Figure 1B). In undifferentiated ES cells, a low level of the long Xist RNA isoform was transcribed in both male and female cells. Upon embryoid body (EB) differentiation, robust *Xist* upregulation was induced in female cells but lower *Xist* expression was maintained in male cells. Finally, whereas *Xist* expression was extinguished in male mouse embryonic fibroblast (MEF) cells, robust *Xist* expression was maintained in female MEF cells. Surprisingly, the short splicing isoform of Xist RNA was detected not only in female ES and EB cells but also in male ES and EB cells (Figure 1B).

To determine the relative expression level of short and long splicing isoforms of *Xist*, we first determined the amplification efficiencies of short and long splicing isoform-specific primer pairs (Figure 1C). Ten-fold serial dilutions of genomic DNA extracted from the heterozygous *Xist* intron 7-deletion mutant female ES cells were used as templates for quantitative real-time PCR analysis (See Supplementary Figure S2). This *Xist* mutant female ES cell line has one wildtype X chromosome with the full length of *Xist* and the other mutant X chromosome deleted a 5.7-kb region corresponding to *Xist* intron 7 of the short splicing isoform. The standard curves created by the qRT-PCR data showed that both short and long splicing isoform-specific primer pairs amplified the target region with high efficiency (91% and 93%, respectively). We then examined the ratio of short and long splicing isoforms of Xist RNA expressed in different cell types (Figure 1D). Compared with the long splicing Xist isoform, the short splicing Xist RNA isoform was expressed at a lower level in all types of cells we tested except for male MEF cells which do not express *Xist*. Interestingly, male cells showed a higher short Xist isoform ratio than female cells. The ratio of the short splicing Xist isoform against the long isoform was approximately 0.30 in undifferentiated ES and 0.20 in differentiating EB male cells, respectively. On the other hand, in all types of female cells, the ratio of the short splicing Xist RNA isoform was consistent at ∼0.05.

### CRISPR/Cas9-based modulation of splicing efficiency for *Xist* intron 7

**Figure 2.**
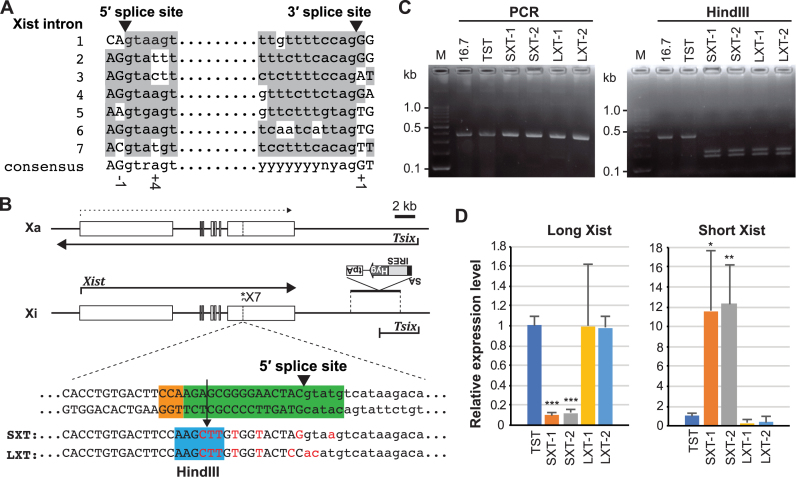
Short and long Xist isoform-specific targeting with CRISPR/Cas9. (**A**) Alignment of all 5′ and 3′ splice sites in *Xist* with a consensus splicing sequence of a major U2 class intron (36,37). r: adenine (a) or guanine (g); y is cytosine (c) or thymine (t). The exon and intron nucleotide sequences are capitalized or lowercased, respectively. Arrowheads indicate cleavage sites by splicing. (**B**) Map of *Xist/Tsix* locus to show the strategy of isoform-specific targeting and the *Tsix* truncation. The asterisk indicates the position of primer pair (X7) used in (C). The sgRNA sequence and adjacent protospacer-adjacent motif (PAM) for CRISPR/Cas9 genome editing are shown as green and orange boxes, respectively. The mutations introduced by CRISPR/Cas9 are labeled in red. Arrow indicates double-strand break site. HindIII site to confirm CRISPR/Cas9-based targeted HDR is highlighted by blue. SA, splicing acceptor; IRES, internal ribosome entry site; Hyg, hygromycin resistance gene; tpA, tandem polyadenylation signal. (**C**) Genomic PCR and following HindIII digestion analysis to confirm the CRISPR/Cas9 targeting. (**D**) qRT-PCR of short and long *Xist* expression in ES cells to confirm the splicing efficiency alteration in isoform-specific targeting cell lines. *Gapdh* was used as an internal control for normalization. The mean ± SD from three independent experiments is shown. P-values were calculated to TST control by an unpaired t-test (*P < 0.05, **P < 0.01, ***P < 0.001).

Given the short isoform of Xist RNA lacks the hnRNP U binding region which presents in exon 7 of the long splicing Xist isoform (Figure 1A) (20), we next sought to address whether the short isoform of Xist RNA functions to induce XCI: stable localization of Xist RNA and induction of X-linked gene silencing on the Xi. Although it remains difficult to predict alternative splicing efficiency from DNA sequences at the 5′ and 3′ splice sites, it is worth noting that the 5′ and 3′ splice sites of alternative splicing often differ from consensus sequence in constitutive splicing (2). Thus, the 5′ and 3′ splice sites could be primary targets for modifications whereby efficiency of alternative splicing is enhanced or repressed. We compared the nucleotide sequences of the 5′ and 3′ splice sites of *Xist* intron 7 with consensus sequences of constitutive splicing sites (36,37), as well as those of the other *Xist* introns, to determine whether modification of the 5′ and 3′ splice sites could alter splicing efficiency of *Xist* intron 7 (Figure 2A) (38). Invariant sequences at the 5′- and 3′-ends of the intron are conserved in all *Xist* introns: GT and AG, respectively (Figure 2A). Three nucleotides in *Xist* intron 7 splice sites are different from the constitutive splicing consensus sequences: –1 and +4 positions of the 5′ splice site, and +1 position of the 3′ splice site of *Xist* intron 7. Considering that a presence of T at position +4 of the 5′ splice site of *Xist* intron 2 and T at position +1 of the 3′ splice site of introns 5 and 6 do not impair their splicing efficiency, C at position –1 of the 5′ splice site of *Xist* intron 7 could be a major reason for the weaker splicing efficiency. Thus, we chose the C at position -1 of the 5′ splice site of *Xist* intron 7 as a primary target for modifications to modulate splicing efficiency. Optimization of the 5′ splice site of *Xist* intron 7 could lead to improved splicing efficiency of *Xist* intron 7 and induce higher expression of the short Xist RNA isoform. In contrast, disruption of invariant sequences at the 5′-end of *Xist* intron 7 is likely to result in no splicing of *Xist* intron 7 and exclusive expression of the long splicing Xist RNA isoform.

To modify the 5′ splice site of *Xist* intron 7 to modulate splicing efficiency for *Xist* intron 7, we used the CRISPR/Cas9-mediated homology-directed repair (HDR) system to introduce mutations using a single-stranded oligodeoxynucleotides (ssODNs) as a repair template (Figure 2B) (39). For CRISPR/Cas9-mediated modification at the 5′ splice site of *Xist* intron 7, we chose a 20 bp-length sequence from position +5 to -15 of the 5′ splice of *Xist* intron 7 as sgRNA sequence. To improve splicing efficiency of *Xist* intron 7 which resulted in dominant expression of the short splicing Xist isoform (termed SXT, short Xist transcript), C to G transversion at position -1 of the 5′ splice site of *Xist* intron 7 was introduced as well as T to A at position +4. In contrast, to abolish *Xist* intron 7 splicing for exclusive expression of the long splicing Xist isoform (termed LXT, long Xist transcript), we used ssODN with mutations in invariant sequences at positions +1 and +2 of the 5′ splice site. In addition, we introduced HindIII site (position –16 to –11 of the 5′ splice site) to easily identify the *Xist* splicing mutant ES clones.

A *Tsix*-truncated 16.7 female ES cell line (TST) was used as a parental cell line to target *Xist* intron 7 in this study. The 16.7 ES cell line has one X-chromosome from *Mus musculus* 129Sv/J (129) and the other from *M. musculus castaneus* (Cast). In the TST cell line, the 129 X with the *Tsix* mutation always become the Xi upon differentiation due to disruption of *Tsix*, a negative regulator of *Xist* (27,40,41), which allows allele-specific analysis of X-linked gene expression. By screening using genomic PCR followed by HindIII digestion, several homozygous *Xist* splicing mutant cell lines were isolated (Figure 2C). We also confirmed the alteration of sequences around the 5′ splice site of *Xist* intron 7 in each cell line by Sanger sequencing (Supplementary Figure S1). To confirm whether the mutations at the 5′ splice site at *Xist* exon 7 alter the splicing efficiency in SXT and LXT *Xist* splicing mutant female ES cells, we analyzed the *Xist* expression by qRT-PCR using short and long isoform-specific primer pairs (Figure 2D). As we predicted, while the expression level of the long Xist RNA isoform was comparable with that in control TST cells, expression of the short Xist isoform was further repressed in LXT mutant cell lines as compared to control TST ES cells. By contrast, in SXT female ES cell lines, the expression level of the long Xist isoform was significantly reduced with approximately 10% in control TST cells whereas the expression level of the short Xist RNA isoform was more than 10 times higher than in TST cells. These data indicate that SXT and LXT mutations at the 5′ splice site of *Xist* intron 7 by CRISPR/Cas9-mediated HDR can efficiently modulate splicing efficiency of *Xist* intron 7.

We also created female ES cell lines dominantly expressing long and short splicing isoform of *Xist* from 129 X-chromosome by traditional gene targeting (Supplementary Figure S2). Using successive gene targeting followed by cre-loxP recombination, we replaced 5′ and 3′ splice sites of *Xist* intron 7 by loxP, yielding female ES cells expressing long splicing Xist isoform with 2 loxP sites. In addition, we also obtained female ES cell lines expressing short splicing Xist isoform with 1 loxP. Finally, we disrupted *Tsix* to induce non-random XCI from 129 mutant *Xist* allele. Compared with CRISPR/Cas9-based approach, the strategy using traditional gene targeting to generate short splicing Xist isoform-expressing ES cells is associated with large deletion of genomic sequence from *Xist*. Another disadvantage of traditional gene targeting for our *Xist* mutant ES cells is that series of gene targeting and Cre-loxP recombination took more than 4 months compared with CRISPR/Cas9-based approach which took approximately one month. Since our *Xist* mutant female ES cell lines created by gene targeting exhibited similar phenotype to those created by CRISPR/Cas9-based approach in *Xist* expression and X-linked silencing upon differentiation, results obtained from *Xist* mutant ES cell lines by traditional gene targeting are shown in Supplementary data (Supplementary Figures S2 and S4).

This CRISPR/Cas-mediated modulation can be also used to modulate other kinds of alternative splicing events. As an example, we modified the efficiency of *Tsix* exon 3 skipping (Supplementary Figure S3). Optimization of the 3′ splicing site in *Tsix* intron 2 significantly enhanced the efficiency of exo3-incorporation in the Tsix transcript.

### *Xist* upregulation occurs normally in SXT and LXT female cells

**Figure 3.**
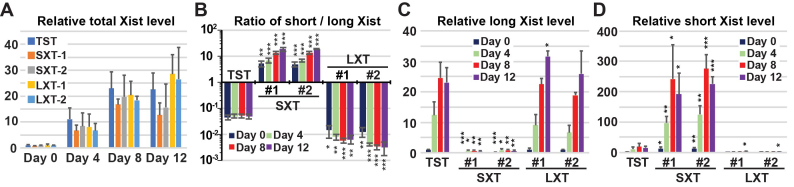
*Xist* upregulation in isoform-specific targeting cells during ES cell differentiation. (**A**) 129 Xi allele-specific qRT-PCR of the *Xist* expression across exons 1–3. (**B**) Ratio of short splicing isoform of Xist RNA to long Xist isoform in control TST and *Xist* splicing mutant cells upon differentiation. Xist splicing isoform-specific primer pairs (XL and XS in Figure 1A) were used for qRT-PCR. (**C, D**) Expression of long (C) and short (D) splicing isoform of Xist RNA using qRT-PCR with Xist splicing isoform-specific primer pairs (XL and XS). The expression values in (A), (C), and (D) were normalized to *Gapdh* and those of the undifferentiated control TST cells which is set to 1. The mean ± SD from three independent experiments is shown. *P*-values were calculated to TST control at the same day of differentiation by an unpaired *t*-test (*P < 0.05, **P < 0.01, ***P < 0.001).

We next examined the effects of SXT and LXT mutations on *Xist* upregulation when we differentiated *Xist* splicing mutant female cells to induce non-random XCI due to *Tsix* mutation. To measure the total *Xist* expression level from the 129 Xi, we performed 129 mutant allele-specific qRT-PCR analysis to determine total *Xist* expression from the Xi using the primer pair (X1–3) that spans introns (Figures 1A and 3A and Supplementary Figure S4A). The expression levels of total Xist RNA from the 129 Xi gradually increased during ES cell differentiation in all cell lines. No significant difference in *Xist* upregulation was observed upon differentiation between control TST cells and SXTs or LXTs mutant cells, although the Xist RNA level in SXT mutant cells was slightly lower than that of control TST and LXT mutant cells at day 12 of differentiation. These data suggest that SXT and LXT mutant female ES cell lines can induce *Xist* upregulation even though several nucleotides are replaced at the 5′ splice site of *Xist* intron 7 by CRISPR/Cas9-mediated HDR.

Next, we used qRT-PCR with splicing isoform-specific primer pairs (XL and XS in Figure 1) to examine whether dominant expressions of short- and long-splicing isoforms of Xist RNA in SXT and LXT mutant ES cells (Figure 2D), respectively, are maintained during ES cell differentiation. We determined the ratio of short splicing isoform to long splicing isoform of Xist RNA based on qRT-PCR data using Xist splicing isoform-specific primer pairs (Figure 3B and Supplementary Figure S4B). The proportion of the short Xist RNA isoform in control TST cells was constantly low (approximately 0.05 of short/long splicing Xist isoform ratio) during differentiation, consistent with data in Figure 1D. Dominant expression of the short splicing isoform of Xist RNA in SXT *Xist* splicing mutant cells became more evident as differentiation progressed. At day 12 of differentiation, short splicing isoform of Xist RNA was approximately 20-fold higher than long Xist isoform (Figure 3B). Throughout ES cell differentiation, the relative expression level of the long Xist splicing isoform in SXT mutant cell lines was much lower than that in undifferentiated control TST cells (Figure 3C). In contrast, in LXT *Xist* splicing mutant cells, short isoform *Xist* expression was significantly lower (approximately 0.01 or less) than those in TST cells, which exhibited approximately 0.05 short/long Xist isoform ratio constantly during ES cell differentiation (Figure 3B). During ES cell differentiation, LXT mutant cell lines consistently exhibited weaker expression of the short Xist isoform than undifferentiated control TST cells (Figure 3D). This data suggested that the CRISPR/Cas9-mediated SXT and LXT mutations at the 5′ splice site of *Xist* intron 7 result in dominant expression of short and long splicing isoforms of Xist RNA, respectively.

### Short Xist splicing isoform coats Xi and recruits repressive histone modifications to the Xi

**Figure 4.**
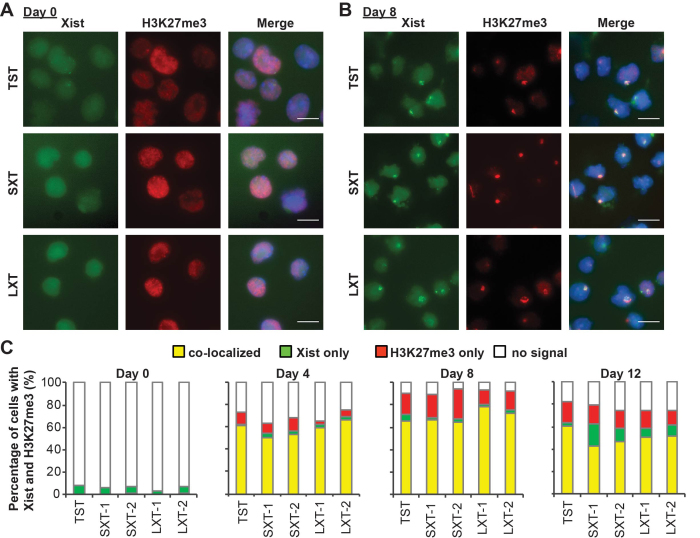
Accumulation of Xist RNA and H3K27me3 on the Xi by short and long splicing mutant Xist RNA. (**A** and **B**) Representative image of Immuno-FISH for Xist RNA (green) and H3K27me3 (red) in undifferentiated ES cells (A, day 0) and differentiating EB cells (B, day 8). Nuclei were counterstained with DAPI. Scale bar is 10 μm. (**C**) Frequency of Xist RNA cloud- and H3K27me3-positive cells upon differentiation. More than 200 nuclei were counted at each time point for each cell line in three independent experiments.

One of the characteristic features of Xist RNA is its unique localization along the entire Xi to induce X-linked gene silencing (42). Nuclear scaffold protein hnRNP U is a critical factor for Xist RNA to associate with the Xi (23,43). Since the short splicing isoform of Xist RNA lacks one of the two major hnRNP U binding region in exon 7 of long Xist splicing isoform (20), we examined whether the short isoform of Xist RNA can be localized on the Xi and recruit histone modifications enzymes to the Xi. We differentiated *Xist* splicing mutant cell lines, as well as control TST cells, and performed immunofluorescence against histone H3 trimethyl lysine 27 (H3K27me3) combined with fluorescence in situ hybridization (FISH) for Xist RNA (Figure 4). At the undifferentiating stage (day 0), neither Xist RNA clouds nor focal H3K27me3 signal was observed in the nucleus in both SXT and LXT *Xist* mutant cells as well as control TST cells (Figure 4A and C). Upon differentiation, the percentage of cells with Xist RNA clouds and focal H3K27me3 enriched on the Xi was increased in all cell lines (Figure 4B and C). The percentage of Xist-positive and H3K27me3-positive cells was comparable between TST cells and *Xist* splicing mutant cells (SXT and LXT) at each stage during ES cell differentiation. Consistent with a previous report that *Xist* exon 7 is not required for PRC2 recruitment (20), these results demonstrate that *Xist* intron 7 is dispensable for PRC2 recruitment to the Xi. These data also indicate that the short splicing isoform of Xist RNA as well as the long isoform is able to localize on the Xi.

Since Xist RNA recruits various chromatin modifying enzymes to the Xi to induce chromosome-wide gene silencing (9,10), we examined the deposition of other histone modifications on the Xi in SXT and LXT mutant female cells upon differentiation. We performed immunofluorescence for two epigenetic hallmarks of the Xi, ubiquityl-histone H2A (H2AK119Ub) and histone H4 monomethyl lysine 20 (H4K20me1) (Supplementary Figure S5). Similar to the staining pattern of H3K27me3, no focal signal of H2AK119Ub and H4K20me1 was evident in undifferentiated female ES cells. However, focal stainings of these histone marks were colocalized with H3K27me3 on the Xi upon differentiation. The staining pattern of these histone marks showed no significant difference between control cells and *Xist* splicing mutant cells during X-inactivation. Consistent with our recent study showing that *Xist* repeat E is required for ASH2L localization to the Xi (21), ASH2L recruitment to Xi was normal in both SXT and LXT *Xist* splicing mutant cell lines since both SXT and LXT mutant Xist RNAs retains *Xist* repeat E (Supplementary Figure S6). No significant difference was found between control and *Xist* splicing mutant cells at any stage. These data suggest that the short isoform of Xist RNA can recruit various histone modifying enzymes and chromatin factors to establish unique heterochromatic features on the Xi.

**Figure 5.**
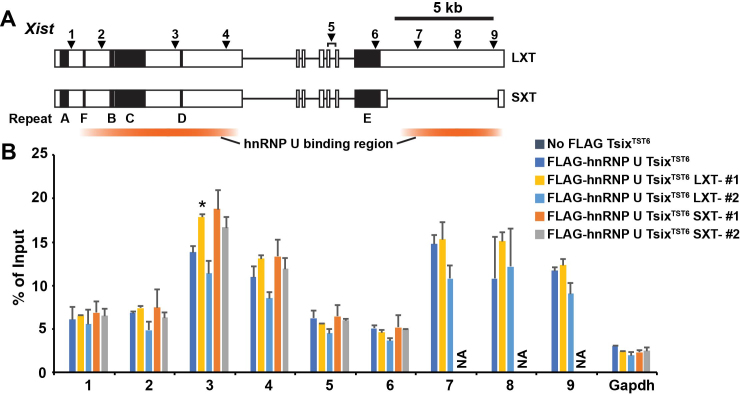
Interaction of short splicing Xist isoform with hnRNP U through exon 1. (**A**) Map of primer pairs across *Xist* for the RIP analysis. White boxes indicate *Xist* exons. The *Xist* repeats A-F are shown by black boxes. The positions of the primer pairs across *Xist* are shown as arrowheads. (**B**) UV- crosslinking RIP analysis using *Xist* splicing mutant female ES cell lines expressing FLAG-HA-tagged hnRNP U upon differentiation at day 9. The mean ± SD bar from two independent experiments is shown with an unpaired *t* test *P* values (*P < 0.05).

Since *Xist* intron 7 region contains one of two hnRNP U binding regions present in long splicing Xist isoform (Figure 5A) (20), we next addressed whether hnRNP U-binding to Xist RNA is maintained in the short splicing Xist isoform by UV-crosslinking RNA immunoprecipitation (RIP). Using the FLAG-HA-hnRNP U *Tsix^TST6^* female ES cell line, which expresses FLAG-hnRNP U from endogenous loci and has *Tsix* truncation mutation on 129 allele (20), we established SXT and LXT *Xist* mutant cell lines by CRISPR/Cas9-based approach (Supplementary Figure S7). Compared with control TST and LXT mutant cell lines, UV-crosslinking RIP using anti-FLAG antibody revealed that hnRNP U-binding to *Xist* exon 1 is comparable in SXT *Xist* mutant cell lines that dominantly express short splicing Xist isoform (Figure 5B). This result indicates that short splicing Xist isoform is able to bind with hnRNP U though exon 1 as efficiently as long splicing Xist isoform.

### Short Xist isoform is sufficient to induce X-linked gene silencing on the Xi

**Figure 6.**
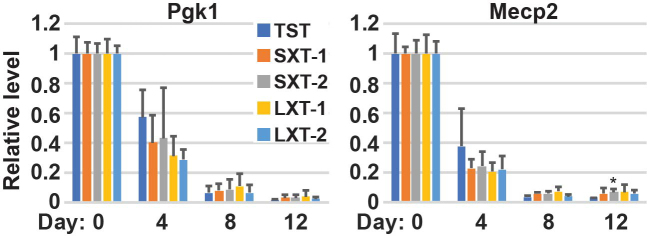
X-linked gene silencing induced by Xist short and long isoform. qRT-PCR using 129 Xi allele-specific primer sets for two Xi-linked genes, *Pgk1* and *Mecp2. Gapdh* was used as an internal control for normalization. Each value was also normalized to that of each undifferentiated cell line. The mean ± SD from three independent experiments is shown. *P*-values were calculated by an unpaired t-test (*P<0.05).

Since differentiated SXT mutant female cells expressing a robust short isoform of Xist RNA exhibited normal localization to the Xi and accumulation of various histone modifications on the Xi similar to control TST cells (Figures 3 and 4), we next examined whether the short splicing isoform of Xist RNA can normally induce X-linked gene silencing during XCI. We performed qRT-PCR analyses using 129 Xi allele-specific primer pairs for two X-linked genes, *Pgk1* and *Mecp2* (Figure 6 and Supplementary Figure S4C and S4D). Upon ES cell differentiation, the levels of X-linked genes decreased significantly from day 0 to day 12 in control TST cells, as expected. In both SXT and LXT cell lines, expression levels of *Pgk1* and *Mecp2* genes from 129 Xi allele were gradually decreased with no significant difference from control TST (Figure 6). Although silencing of *Mecp2* slightly preceded *Pgk1* silencing, both X-linked genes were efficiently repressed less than 8% at day 12 than those in undifferentiated ES cells (day 0). These qRT-PCR data for X-linked genes indicate that both short and long Xist isoforms can induce X-linked gene silencing during XCI comparable to control TST cell line. In sum, despite missing a large region from the long splicing Xist isoform, the short splicing isoform of Xist RNA functions as efficiently as the long splicing isoform of Xist RNA in XCI.

## DISCUSSION

In this study, we provide a CRISPR/Cas-based strategy to create cell lines expressing specific splicing isoforms of Xist RNA. The alternative splicing of *Xist* intron 7 belongs to a class of intron retention. Although intron retention has been described as a consequence of mis-splicing, recent studies show that intron retention is involved in regulation of protein-coding gene expression through nuclear retention, exosome degradation or nonsense-mediated mRNA decay (44,45). Due to the complexity of alternative splicing, in which many DNA elements (multiple 5′ and 3′ splice sites, splicing enhancers, splicing silencers etc.) are involved (46), it is still difficult to predict splicing efficiency of alternative splicing to determine the targeted modification to impact splicing efficiency. However, in terms of the numbers of the 5′ and 3′ splice sites involved, intron retention is the simplest alternative splicing class in which only one 5′ and one 3′ splice sites are involved. Although invariant sequences at the 5′ splice site (position +1 and +2) in *Xist* intron 7 are conserved, we found that highly conserved G in constitutive intron at position –1 of the 5′ splice site is replaced by C in *Xist* intron 7. We suspected that this nucleotide change causes less efficient splicing of *Xist* intron 7 and chose the 5′ splice site to enhance or repress the retention of *Xist* intron 7 by modulating splicing efficiency using the CRISPR/Cas-based strategy. This approach provides a platform of genome editing to study functions of specific splicing isoforms of a transcript generated by intron retention, which is found in transcripts from approximately three quarters of multi-exonic genes in mice and humans (47,48). Untranslated regions and noncoding RNAs have retained introns more frequently than protein-coding regions. In addition, we also show that we can modulate the efficiency of exon-skipping type alternative splicing in *Tsix* by CRISPR/Cas-mediated intron targeting (Supplementary Figure S3). Our described method does not require complex construction of targeting vectors; as such, our method provides a useful approach to study functions of various alternatively spliced transcripts.

Our RT-PCR analysis of the short splicing isoform of Xist RNA differs from a previous report (16). The previous report showed that the short splicing isoform of Xist RNA is exclusively expressed in female cells but not in male (16). In our qRT-PCR experiments (Figure 1), we detected short Xist isoform expression in both male and female cells, and the ratio of the short Xist isoform in male undifferentiated ES and differentiating EB cells to the long isoform is even higher than that in female cells, although the level of the short splicing isoform was lower than the long Xist splicing isoform. It is possible that culture conditions affect the alternative splicing pattern of *Xist* in male ES and EB cells. Alternatively, differences in relation to expression of the short splicing isoform of Xist RNA could result from the different male ES cell lines used in the previous report and our work. Although the short splicing Xist isoform is expressed in male ES and EB cells, *Xist* expression level is constantly low and is finally extinguished in fully differentiated MEFs. Thus, it is not likely that short splicing isoform of Xist RNA plays a crucial function in Xist RNA-mediated X-linked gene regulation in male cells.

**Figure 7.**
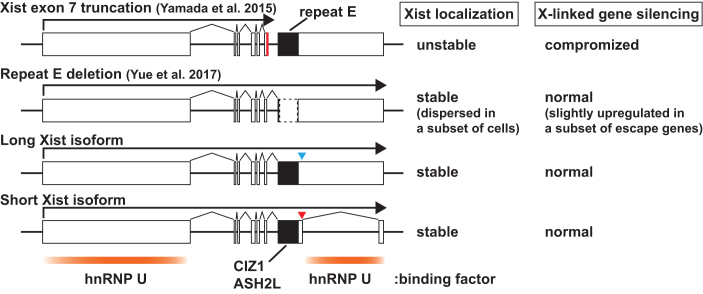
Summary of phenotypes in *Xist* exon 7 mutant female ES cell lines. White and black rectangles indicate *Xist* exon and repeat E elements, respectively. Red line shows polyadenylation signal inserted to truncate *Xist* exon 7 (20). Dotted rectangle indicates *Xist* repeat E deletion (21). Blue and red arrowheads show mutations at the 5′ splice site in *Xist* intron 7 to disrupt and enhance splicing of intron 7, respectively. hnRNP U binding regions are shown below *Xist* mutant maps.

Despite its low expression level, the short Xist isoform is consistently expressed in all types of female cells (Figure 1D). Whereas exon 7 in the long Xist isoform contains one of the two major hnRNP U binding regions (20), the short splicing isoform of Xist RNA loses the hnRNP U binding region present in exon 7 of long Xist isoform by intron 7 splicing (Figure 7). Since hnRNP U is a critical protein factor for Xist RNA localization on the Xi (23), we sought to examine whether the short Xist isoform can induce XCI using *Xist* splicing SXT mutant female ES cells expressing the short isoform of Xist. To our surprise, SXT mutant cell lines exhibit normal Xist RNA localization on the Xi and X-linked gene silencing (Figures 4 and 6). Since *Xist* exon 7 truncation mutant female ES cells result in unstable localization and compromised X-linked gene silencing upon differentiation (20), 1,658-bp exon 7 including 1.3-kb *Xist* repeat E and 205-bp exon 8 in short splicing isoform of Xist RNA is enough to maintain stable localization of Xist RNA on the Xi as efficiently as the full length of exon 7 in the long Xist isoform. Although *Xist* repeat E deletion does not significantly affect Xist RNA localization, dispersed Xist RNA localization was observed in a subset of *Xist* repeat E mutant female ES cells upon differentiation (21). Furthermore, a nuclear matrix binding protein CIZ1 binds to Xist RNA repeat E and is required for Xist RNA localization on the Xi in a tissue-specific manner (22,49). Thus, various protein factors such as hnRNP U and CIZ1 might have a redundant function through the *Xist* intron 7 region and repeat E to support Xist RNA association with the Xi.

The short splicing isoform of Xist RNA functionally induces XCI in mouse female ES cells upon differentiation (Figures 4 and 6). However, the fact that the short isoform of Xist is maintained at a low expression level in all types of female cells might indicate that the short splicing isoform of Xist is not fully comparable to the long splicing isoform of Xist in a cell-type-dependent or tissue-specific manner. In addition to hnRNP U, variable factors are involved in Xist RNA targeting to the Xi as indicated by recent studies (22,43,49). With low expression of some redundantly acting factors for Xist RNA targeting to the Xi, the hnRNP U binding region present within exon 7 of the long Xist isoform might be more critical than in the differentiating female EB cells used in this study. Further studies are required to fully understand the role of short and long splicing isoforms of Xist during XCI using an in vivo mouse system.

## Supplementary Material

Supplementary Data
