# Supplementary material for: CRISPR/Cas9-mediated modulation of splicing efficiency reveals short splicing isoform of Xist RNA is sufficient to induce X-chromosome inactivation
Source: Nucleic Acids Res. 2017 Dec 9;46(5):e26. doi: 10.1093/nar/gkx1227 (PMC5861412; doi:10.1093/nar/gkx1227)
Supplement: Supplementary Data [file gkx1227_supp.pdf]

Supplementary data

CRISPR/Cas9-mediated modulation of splicing efficiency reveals short splicing isoform of Xist RNA is sufficient to induce X-chromosome inactivation

Minghui Yue[1,2], and Yuya Ogawa[1,2]*

1 Division of Reproductive Sciences, Perinatal Institute, Cincinnati Children's Hospital Medical Center, Cincinnati, Ohio, United States of America, **2** Department of Pediatrics, University of Cincinnati College of Medicine, Cincinnati, Ohio, United States of America

***To whom correspondence should be addressed.** E-mail: yuya.ogawa@cchmc.org

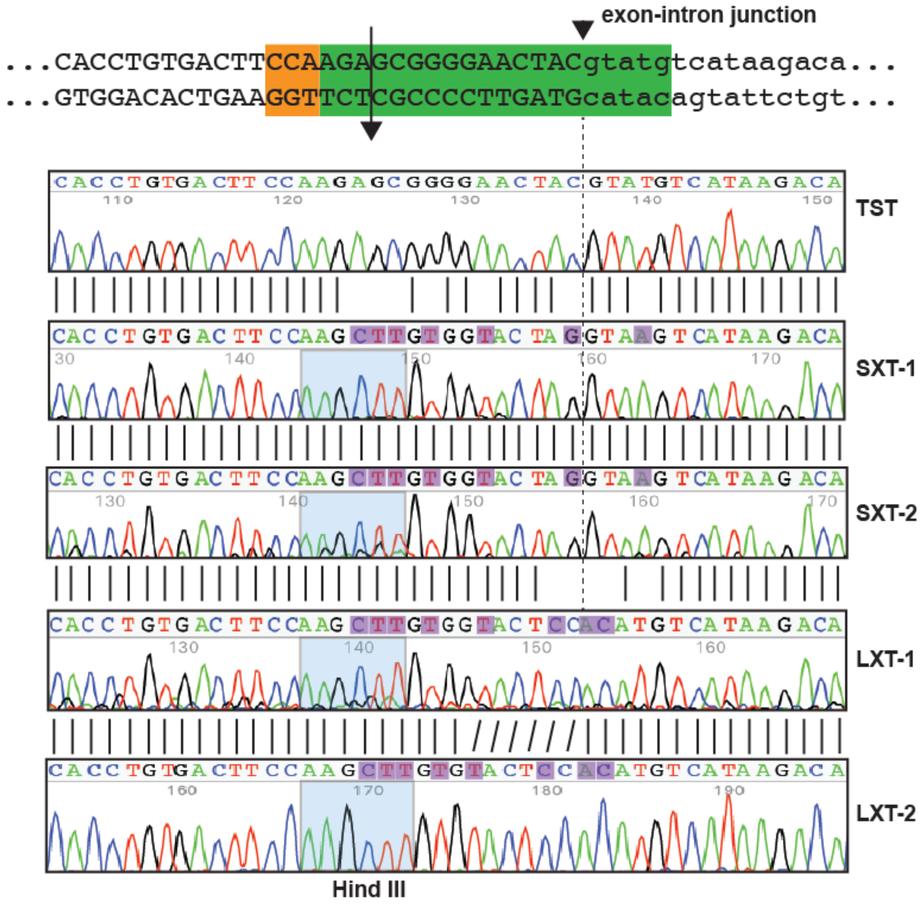

Figure S1. CRISPR/Cas9-mediated targeted modifications at the 5′ splice site of Xist intron 7. Sanger sequencing data of the 5′ splice site in Xist intron 7 in each SXT and LXT mutant cell line. The sgRNA sequence and adjacent protospacer-adjacent motif (PAM) for CRISPR/Cas9 genome editing are shown as green and orange boxes, respectively. Arrow indicates double-strand break site by CRISPR/Cas9. Arrowhead indicates exon-intron junction. Blue highlighted nucleotides showed the HindIII site introduced by CRISPR/Cas9-mediated HDR. Nucleotide positions mutated by CRISPR/Cas9-mediated HDR are highlighted by purple. In LXT-2 mutant cell line, an unexpected deletion of G at position -7 of the 5′ splice site was generated, but it didn’t affect the repression of splicing of Xist intron 7.

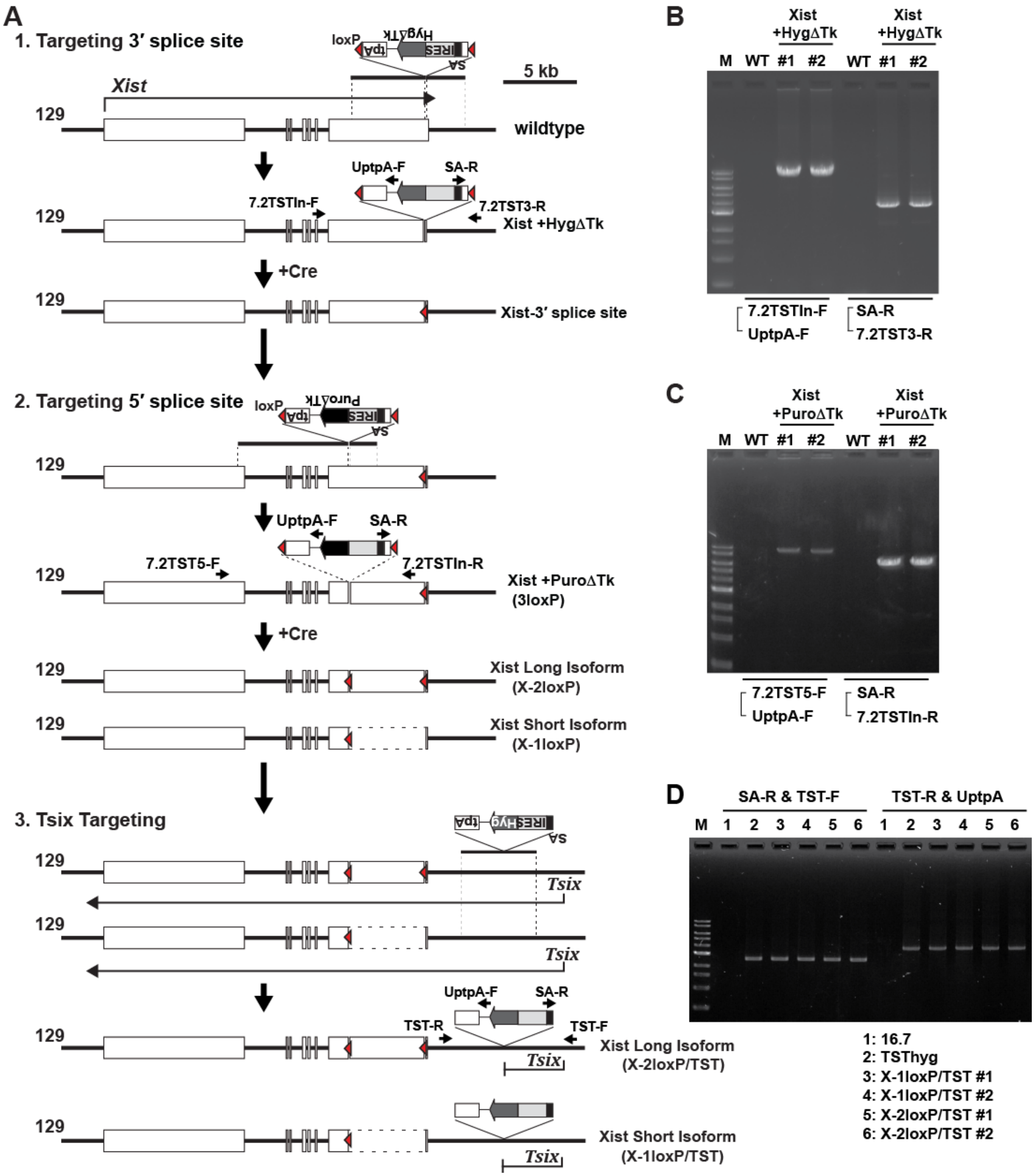
A
1. Targeting 3′ splice site
loxP
5 kb
Xist
129
wildtype
UptpA-F
SA-R
7.2TSTIn-F
7.2TST3-R
Xist +HygΔTk
+Cre
Xist-3′ splice site
2. Targeting 5′ splice site
UptpA-F
SA-R
7.2TST5-F
7.2TSTIn-R
Xist +PuroΔTk
(3loxP)
Xist Long Isoform
(X-2loxP)
Xist Short Isoform
(X-1loxP)
3. Tsix Targeting
Tsix
TST-R
TST-F
Xist Long Isoform
(X-2loxP/TST)
Xist Short Isoform
(X-1loxP/TST)
B
Xist +HygΔTk
M WT #1 #2 WT #1 #2
7.2TSTIn-F
UptpA-F
SA-R
7.2TST3-R
C
Xist +PuroΔTk
M WT #1 #2 WT #1 #2
7.2TST5-F
UptpA-F
SA-R
7.2TSTIn-R
D
SA-R & TST-F
TST-R & UptpA
M 1 2 3 4 5 6 1 2 3 4 5 6
1: 16.7
2: TSThyg
3: X-1loxP/TST #1
4: X-1loxP/TST #2
5: X-2loxP/TST #1
6: X-2loxP/TST #2

Figure S2. Creation of female ES cell lines expressing short or long splicing Xist isoform from the 129 Xi by successive traditional gene targeting. (A) Gene targeting strategy to create female ES cell lines expressing short and long splicing Xist isoform from 129 X-chromosome. SA, splice acceptor. Ires, internal ribosome entry site. tpA, tandem poly(A) signals. Puro∆Tk, puromycin resistance gene-truncated thymidine kinase. Hyg∆Tk, hygromycin resistance gene-∆TK. (B and C) Genomic PCR analysis to confirm the replacement of the 3′ and 5′ splice site of Xist intron 7 by selection cassette flanked with 2 loxP sites, respectively. (D) Genomic PCR analysis to confirm the insertion of gene-trap cassette into downstream of major *Tsix* transcription start site, which results in non-random XCI of the mutant 129 X-chromosome.

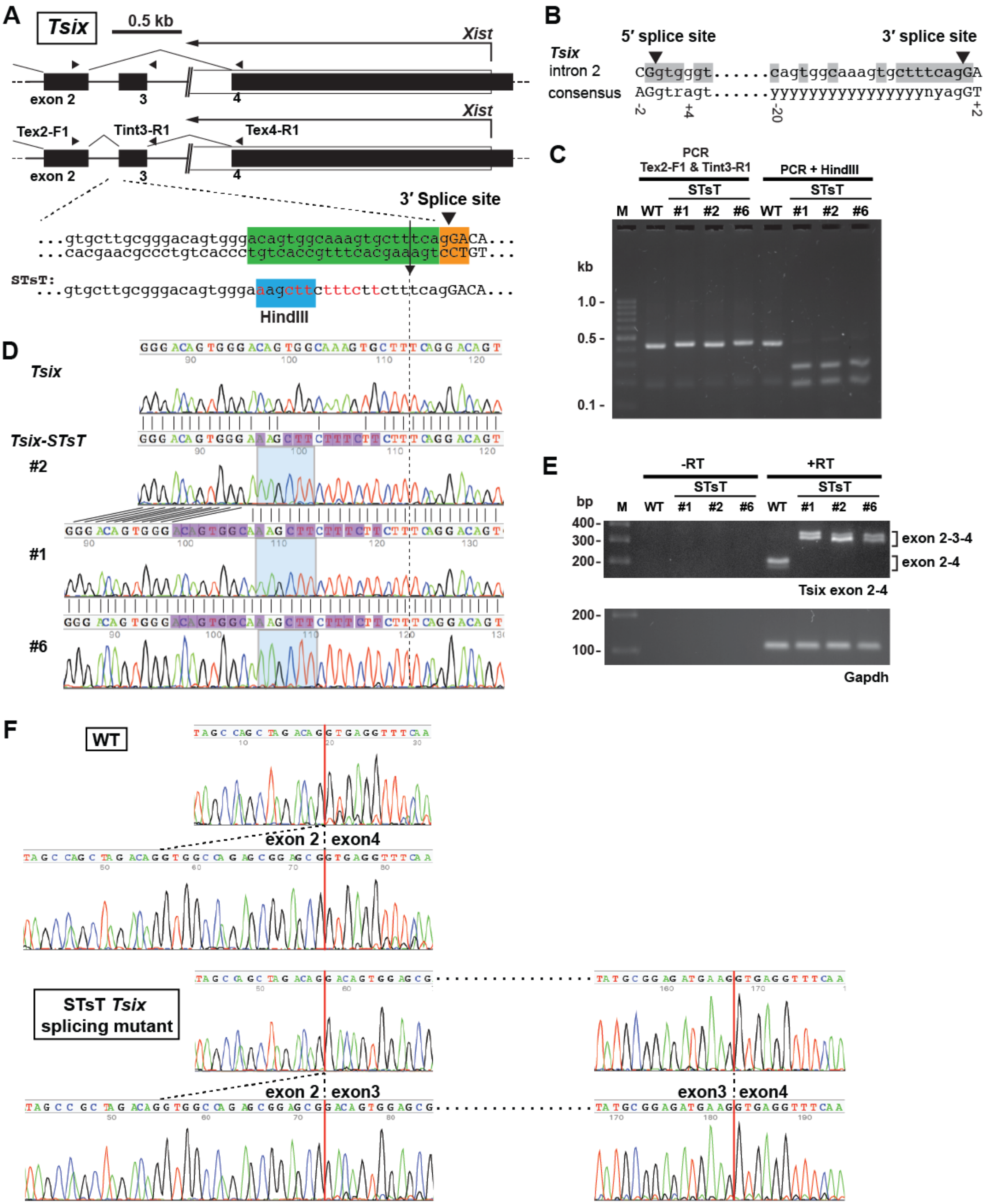
A
Tsix
0.5 kb
Xist
exon 2
3
4
Tex2-F1
Tint3-R1
Tex4-R1
3' Splice site
...gtgcttgcgggacagtgggacagtggcaaagtgctttcagGACA...
...cacgaacgccctgtcaccctgtcaccgtttcacgaaagtcCTGT...
STsT:
...gtgcttgcgggacagtgggaaagcttctttcttctttcagGACA...
HindIII
B
5' splice site
3' splice site
Tsix
intron 2
CGgtgggt......cagtggcaaagtgctttcagGA
consensus
AGgtragt......yyyyyyyyyyyyyyyyynyagGT
-2
+4
-20
+2
C
PCR
Tex2-F1 & Tint3-R1
PCR + HindIII
STsT
M
WT
#1
#2
#6
kb
1.0
0.5
0.1
D
Tsix
Tsix-STsT
#2
#1
#6
E
-RT
+RT
bp
400
300
200
100
exon 2-3-4
exon 2-4
Tsix exon 2-4
Gapdh
F
WT
exon 2
exon4
STsT Tsix
splicing mutant
exon3
exon4

Figure S3. CRISPR/Cas9-mediated targeted modifications at the 3′ splice site of *Tsix* intron 2. (A) Alternative splicing isoform of *Tsix*. *Tsix* exons 2, 3 and 4 are indicated by closed box. Arrowheads show primers used in genomic PCR and RT-PCR analysis in (C) and (E). The sgRNA and adjacent PAM sequences for CRISPR/Cas9 genome editing are shown as green and orange boxes, respectively. The mutations introduced by CRISPR/Cas9 are labeled in red. Arrow indicates double-strand break site. Blue highlighted nucleotides showed the HindIII site introduced by CRISPR/Cas9-mediated HDR. (B) Alignment of the 5′ and 3′ splice site in *Tsix* intron 2 with a consensus splicing sequence of a major U2 class intron. r: adenine (a) or guanine (g); y is cytosine (c) or thymine (t). The exon and intron nucleotide sequences are capitalized or lowercased, respectively. Arrowheads indicate cleavage sites by splicing. (C) Genomic PCR and following HindIII digestion analysis to confirm the CRISPR/Cas9 targeting. (D) Sanger sequencing data of the 3′ splice site in *Tsix* intron 2 modified by CRISPR/Cas9-mediated HDR. Nucleotide positions mutated by CRISPR/Cas9-mediated HDR are highlighted by purple. #1and #3 STsT *Tsix* mutant clones have 8 nucleotides inserted in the upstream of HindIII site introduced by CRISPR/Cas9-mediated HDR. (E) RT-PCR for *Tsix* expression in *Tsix* splicing mutant ES cell lines using primer pairs across exon 2 to 4. Tsix transcript including exon 3, which is rare in wild-type J1 ES cells, increases significantly in CRISPR/Cas9-targeted *Tsix* intron 2 STsT mutant male ES cell line. (F) Sanger sequencing data of RT-PCR products from J1 and *Tsix* STsT mutant #1 in (E). Alternative 5′ splice site, which locates 17 base upstream of the original site, was also detected.

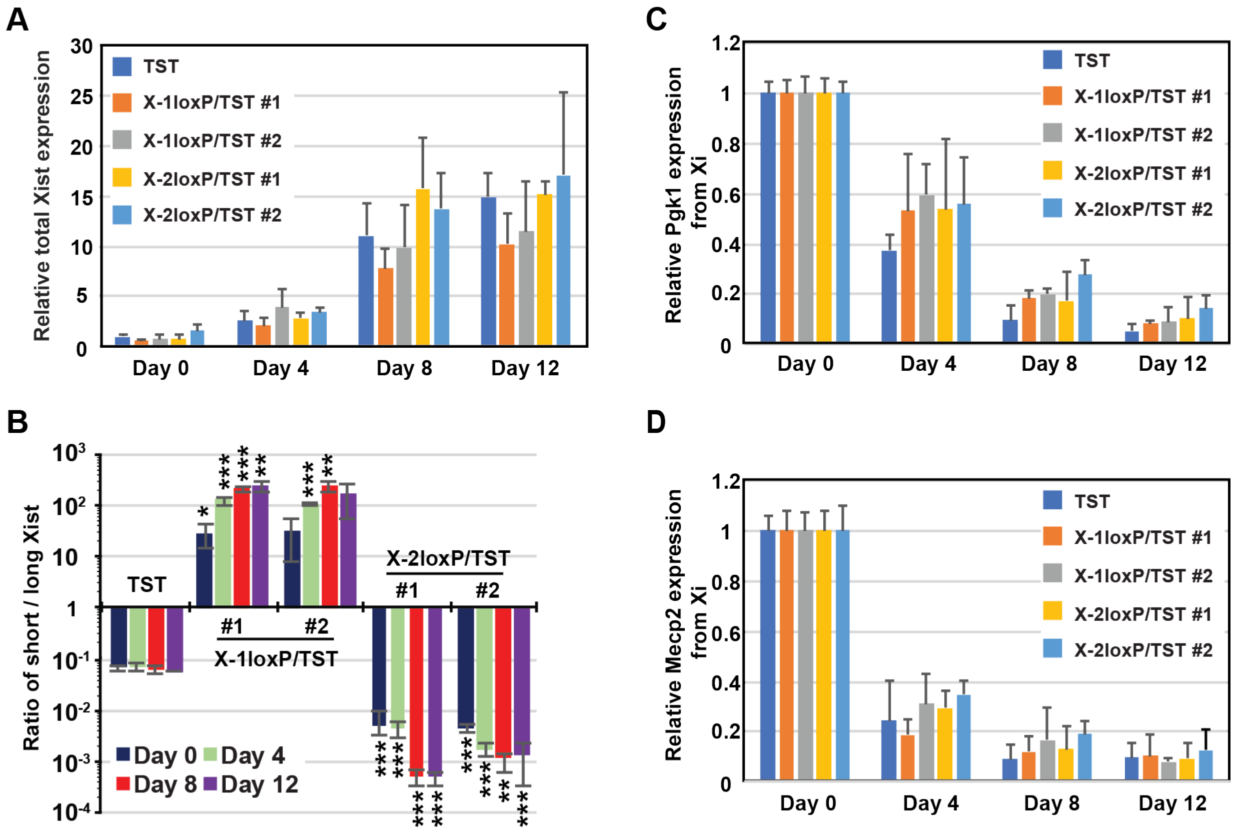

Figure S4. Modulation of short and long splicing isoform *Xist* expression by traditional gene targeting. (A) 129 Xi allele-specific qRT-PCR of total *Xist* expression across exon 1 to 3. Gapdh was used as an internal control for normalization and for those of the undifferentiated control TST cells, which are set to 1. (B) Ratio of short to long splicing Xist isoform in control TST and *Xist* splicing mutant cell lines upon differentiation. Xist splicing isoform-specific primer pairs (XL and XS in Figure 1A) were used for qRT-PCR. (C, D) qRT-PCR using 129 Xi allele-specific primer sets for two Xi-linked genes, Pgk1 (C) and Mecp2 (D). Gapdh was used as an internal control for normalization. Each value was also normalized to that of each undifferentiated cell line. The mean ± SD from three independent experiments is shown. *P*-values were calculated to TST control at the same day of differentiation by an unpaired t-test (*p<0.05, **p<0.01, ***p<0.001).

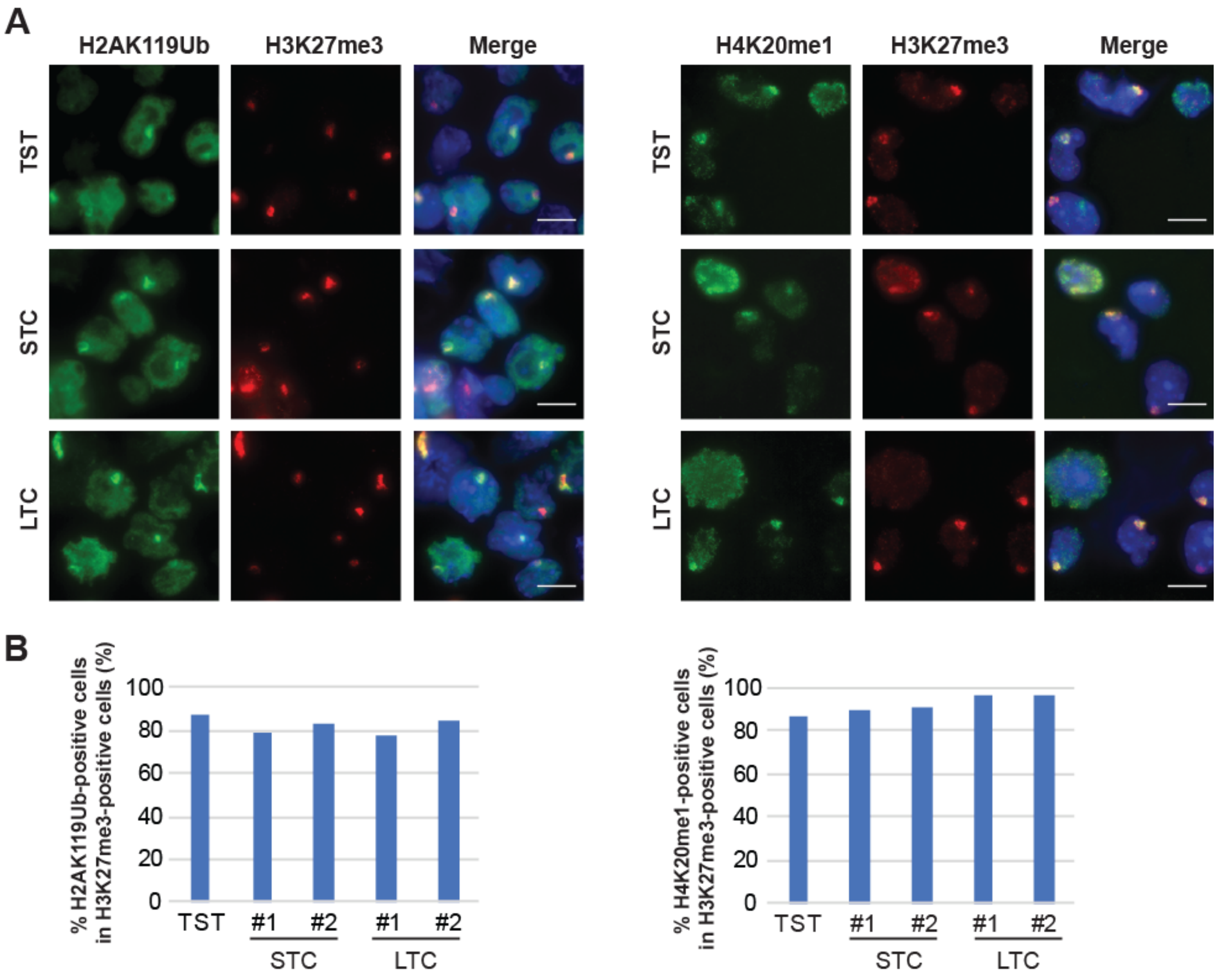

Figure S5. Comparable localization of H2AK119Ub or H4K20me1 and H3K27me3 on the Xi in SXT and LXT mutant cell lines to control TST cell line. (A) immunostaining of H2AK119ub/H4K20me (green) and H3K27me3 (red) at day 8 upon differentiation. Nuclei were counterstained with DAPI. Scale bar is 10μm. (B) Frequency of H2AK119ub- and H4K20me-positive cells with H3K27me3 foci at day 8 of differentiation. More than 100 nuclei were counted for each cell line in two independent experiments.

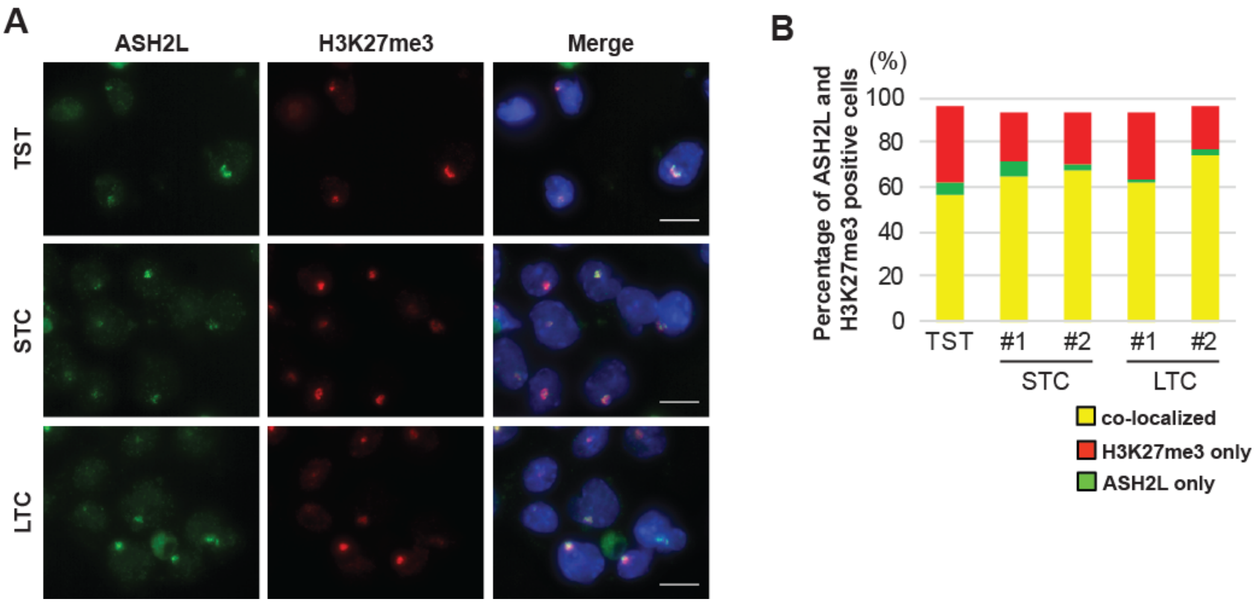

Figure S6. Recruitment of ASH2L to the Xi in SXT and LXT mutant cell lines. (A) immunostaining of ASH2L (green) and H3K27me3 (red) at day 8 upon differentiation. Nuclei were counterstained with DAPI. Scale bar is 10μm. (B) Frequency of ASH2L- and H3K27me3-positive cells at day 8 of differentiation. More than 100 nuclei were counted for each cell line in two independent experiments.

A

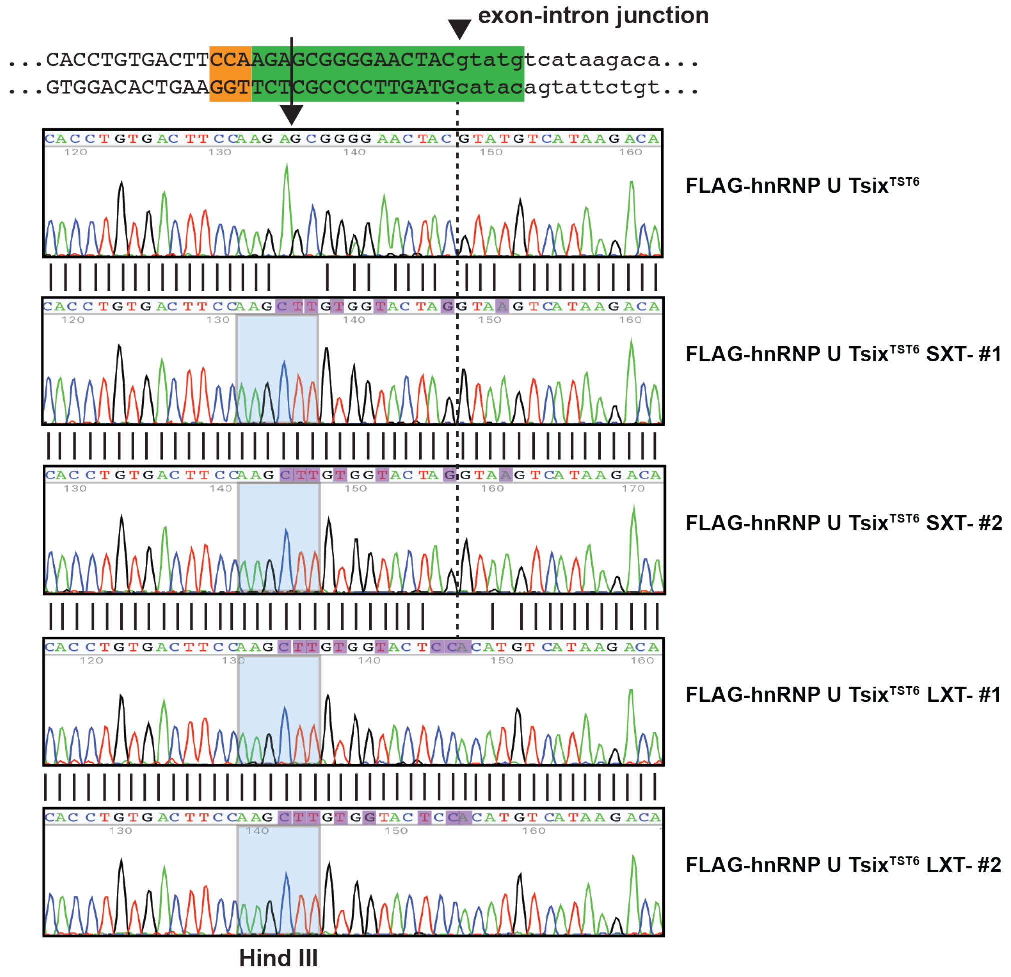
exon-intron junction
...CACCTGTGACTTCCAAGAGCGGGGAACTACgtatgtcataagaca...
...GTGGACACTGAAGGTTCTCGCCCCTTGATGcatacagtattctgt...
FLAG-hnRNP U Tsix[TST6]
FLAG-hnRNP U Tsix[TST6] SXT- #1
FLAG-hnRNP U Tsix[TST6] SXT- #2
FLAG-hnRNP U Tsix[TST6] LXT- #1
FLAG-hnRNP U Tsix[TST6] LXT- #2
Hind III

B

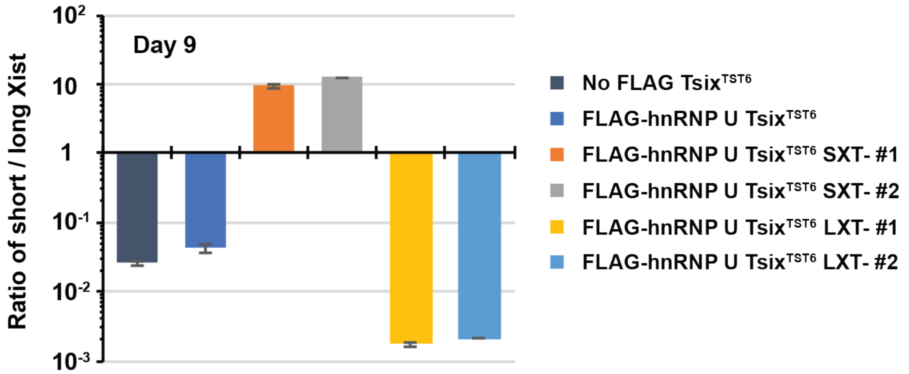
Day 9
Ratio of short / long Xist
10²
10
1
10⁻¹
10⁻²
10⁻³
No FLAG Tsix[TST6]
FLAG-hnRNP U Tsix[TST6]
FLAG-hnRNP U Tsix[TST6] SXT- #1
FLAG-hnRNP U Tsix[TST6] SXT- #2
FLAG-hnRNP U Tsix[TST6] LXT- #1
FLAG-hnRNP U Tsix[TST6] LXT- #2

Figure S7. CRISPR/Cas9-mediated modifications at the 5′ splice site of *Xist* intron 7 in FLAG-hnRNP U/Tsix^{TST6} ES cell line expressing FLAG-HA-hnRNP U. (A) Sanger sequencing data of the 5′ splice site in *Xist* intron 7 in SXT and LXT mutant cell lines expressing FLAG-HA-hnRNP U. The sgRNA and adjacent PAM sequences for CRISPR/Cas9 genome editing are shown as green and orange boxes, respectively. Arrow, double-strand break site by CRISPR/Cas9. Arrowhead, exon-intron junction at the 5′ splice site in *Xist* intron 7. Blue highlighted nucleotides showed the HindIII site introduced by CRISPR/Cas9-mediated HDR. Nucleotide positions mutated by CRISPR/Cas9-mediated HDR are highlighted by purple. (B) Ratio of short to long splicing Xist isoform in control and *Xist* splicing mutant cell lines derived from FLAG-hnRNP U/Tsix^{TST6} cell line. Xist splicing isoform-specific primer pairs (XL and XS in Figure 1A) were used for RT-qPCR.

Supplementary Table S1. Primer information

sgRNA adaptor for Xist intron 7 CRISPR/Cas9 modification
F, CACCGCATACGTAGTTCCCCGCTCT
R, AAACAGAGCGGGGAACTACGTATGC

ssODNs for Xist CRISPR/Cas9 modification
Xist splicing-enhanced,
GGGTCCTTAGGTCTTATGCTTGGACTTAGCTCAGGTTTTGTGTCTTATGACTTACCTAGTACCACAA
GCTTGGAAGTCACAGGTGTCCTGTAGAAACAGTTCCTCTTCTTTGGGTTGT
Xist splicing-repressed,
GGGTCCTTAGGTCTTATGCTTGGACTTAGCTCAGGTTTTGTGTCTTATGACATGTGGAGTACCACA
AGCTTGGAAGTCACAGGTGTCCTGTAGAAACAGTTCCTCTTCTTTGGGTTGT

Xist genomic PCR
Xist-AI7-SD-F, GTCCGGTCAGGATTCAAGTG
Xist-AI7-SD-R, AGAAATGGAAAGGGATGCTG

sgRNA adaptor for Tsix intron 2 CRISPR/Cas9 modification
F, ACCTACAGTGGCAAAGTGCTTTCA
R, AAACTGAAAGCACTTTGCCACTGT

adaptor for tRNA promoter
F,
CATGTGGTTCCATGGTGTAATGGTTAGCACTCTGGACTCTGAATCCAGCGATCCGAGTTCAAATCT
CGGTGGA
R,
AGGTTCCACCGAGATTTGAACTCGGATCGCTGGATTCAGAGTCCAGAGTGCTAACCATTACACCAT
GGAACCA

ssODN for Tsix CRISPR/Cas9 modification
STsT,
GTGCTTGCGGGACAGGGGAGAGGTGGCTAAGTGCTTGCGGGACAGTGGGAAAGCTTCTTTCTTC
TTTCAGGACAGTGGAGCGATGGCTACGTGCTTGCGGGACAGCGGAAGAGATGGTTA

Tsix genomic PCR
Tex2-F1, TCCACGCATCTTGAGTCCTG
Tint3-R1, GCGGCAGGCATTTTAACTATCG

adaptor for gene targeting of 5′ splice site of Xist intron 7

left-arm adaptor
F1, tcgaGAAGAGGAACTGTTTCTACAGGACACCTGTGACTTCCAAGAGCGGGGAACTAC
R1, agctGTAGTTCCCCGCTCTTGGAAGTCACAGGTGTCCTGTAGAAACAGTTCCTCTTC
right-arm adaptor
F2, aattCATAAGACACAAAACCTGAGCTAAGTCCAAGCATAAGACCTAAGGACCCAATCCTATATGc
R2, ggccgCATATAGGATTGGGTCCTTAGGTCTTATGCTTGGACTTAGCTCAGGTTTTGTGTCTTATG

adaptor for gene targeting of 3′ splice site of Xist intron 7
left-arm adaptor
F1, tcgAGTAATATCCCTTTGTATGGGTAGGAATGGGCTTACATTTCTGGAATTTGCAAAGGAAAAAA
R1, agctTTTTTTCCTTTGCAAATTCCAGAAATGTAAGCCCATTCCTACCCATACAAAGGGATATTAC
right-arm adaptor
F2, aaTTACTGCCATCAATATTGCTACCAATTAAGCACATCCTACCATCATCTGC
R2, ggccGCAGATGATGGTAGGATGTGCTTAATTGGTAGCAATATTGATGGCAGT

Genomic PCR for Xist and Tsix targeting
Xist intron 7, 5′ splice site targeting:
5′-end
7.2TST5-F, GTCTCAGTTGCCTTCTCCTTGCTCCCACTG
UptpA-F, CTTTCCGAGGGACACTAGGCTGACTCCAT
3′-end
SA-R, AAACCCTGGACTACTGCGCCCTACAGATCT
7.2TSTIn-R, TCCCAGACCTCTTCAACCTGGCTCCATCTT

Genomic PCR for Xist intron 7, 3′ splice site targeting:
5′-end
7.2TSTIn-F, TCCCCAAAGCAGCACAGAAAACTGGGTCTT
UptpA-F, CTTTCCGAGGGACACTAGGCTGACTCCAT
3′-end
SA-R, AAACCCTGGACTACTGCGCCCTACAGATCT
7.2TST3-R, GGGAGGAGTGGACTCTGCCTCTGTCATTCT

Genomic PCR for Tsix targeting:
5′-end
TST-F, GGAGATCGCTAAAATCCCTGCCTTATAACCAA
SA-R, AAACCCTGGACTACTGCGCCCTACAGATCT
3′-end
TST-R, AATTGGATATCCCTCGCATCTACCTACTTGGAA
UptpA-F, CTTTCCGAGGGACACTAGGCTGACTCCAT

RT-qPCR [ref. (1)]
129 allele-specific Xist (X1-3), $Xist^{129}$-E1-3-F and $Xist^{129}$-E1-3-R (Tm, 62 °C)
129 allele-specific Pgk1, $Pgk1^{129}$-F and $Pgk1^{129}$-R (Tm, 60 °C)
129 allele-specific Mecp2, $Mecp2^{129}$-F and $Mecp2^{129}$-R (Tm, 58 °C)
Gapdh, Gapdh-F and Gapdh-R (Tm, 60 °C)
long Xist isoform (XL), XiI7LRT-F and XiI7LRT-R (Tm, 61 °C)
short Xist isoform (XS), XiI7SRT-F2: ACAGGACACCTGTGACTTCCA and XiI7SRT-R (Tm, 61 °C)

RT-PCR
Tex2-F1, TCCACGCATCTTGAGTCCTG
Tex4-R1, CGGATCCGATTCGAGAGACC
Gapdh-F and Gapdh-R [ref. (1)]

UV-crosslinking RIP [ref. (1)]
1-F, ATCGTTTGGTGCTGTGTGAG
1-R, CTGGCTCGAGAATAGCCGTA
2-F, CGTCTGATAGTGTGCTTTGCTA
2-R, AAGAGTAGCTCGGTGGATGAGT
3-F, CCAATACGGTCAATGGTCCT
3-R, TGAGGAAGGGGTTTCAAGTG
4-F, ATCTTTGCTTGGTCTTTACTACA
4-R, AAAATAAGCAAGGACTGGTGAC
5-F, GAAAGCTTTGCCAGCTGTTT
5-R, ACCCAGTTTTCTGTGCTGCT
6-F, TTGCATGCATCCCTCTCTTT
6-R, AACAGAGAAAGTGGCCCAAG
7-F, CTGCTTTGGTGAGGCTCAGT
7-R, CCCCTTTGTTATTCCCAGTG
8-F, GAGTACGCTGTTGCTGTC
8-R, AGAGTCTGACTGCTCTTC
9-F, GCTGAAGTCACAATTTGCTG
9-R, CTTCAACCTGGCTCCATCT
Gapdh-F, CCTCGTCCCGTAGACAAAATG
Gapdh-R, TCTCCACTTTGCCACTGCAA

References

1. Yamada,N., Hasegawa,Y., Yue,M., Hamada,T., Nakagawa,S. and Ogawa,Y. (2015) Xist Exon 7 Contributes to the Stable Localization of Xist RNA on the Inactive X-Chromosome. *PLoS Genet*, **11**, e1005430.
